# Anodal Transcranial Direct Current Stimulation-Induced Effects Over the Right Dorsolateral Prefrontal Cortex: Differences in the Task Types of Task Switching

**DOI:** 10.3389/fpsyg.2021.630239

**Published:** 2021-03-18

**Authors:** Ziyu Wang, Rongjuan Zhu, Xuqun You

**Affiliations:** Key Laboratory for Behavior and Cognitive Neuroscience of Shaanxi Province, School of Psychology, Shaanxi Normal University, Xi'an, China

**Keywords:** dorsolateral prefrontal cortex (DLPFC), transcranial direct current stimulation (tDCS), task switching, tDCS-induced effect, task-specific effect

## Abstract

Transcranial direct current stimulation (tDCS) has been previously used to investigate the causal relationships between the dorsolateral prefrontal cortex (DLPFC) and task switching but has delivered inconclusive results that may be due to different switching tasks involving different cognitive control processes. In the current study, we manipulated task types and task predictability to investigate the role of DLPFC in task-switching performances. Notably, we distinguished the specific effects of anodal-tDCS on two types of tasks (parity/magnitude and parity/vowel-consonant tasks). Forty-eight participants were randomly assigned to four task groups as follows; Group I who was assigned right anode (RA) parity/magnitude tasks, Group II who were assigned sham parity/magnitude tasks, Group III who were assigned RA parity/vowel-consonant tasks, and Group IV who were assigned sham parity/vowel-consonant tasks. Participants were asked to complete both predictable and unpredictable tasks. In the parity/magnitude task, we demonstrated a lower switch cost for the RA group compared to the sham group for unpredictable tasks. In contrast, in the parity/vowel-consonant task, the switch cost was higher for the RA group compared to the sham group for unpredictable and predictable tasks. These findings confirmed an anodal-tDCS-induced effect over the right DLPFC both in the parity/magnitude and parity/vowel-consonant tasks. Our data indicated that anodal tDCS may have a stronger influence on task-switching performance over the right DLPFC by changing the irrelevant task-set inhibition process. Also, the right DLPFC is unlikely to act by performing exogenous adjustment of predictable task switching.

## Introduction

Task switching is a cognitive process that requires participants to switch between tasks and is often used to study individual cognitive flexibility (Koch et al., 2018). In task-switching paradigms, participants are required to switch in either predictable or unpredictable sequences (Rogers and Monsell, 1995; Meiran, 1996). Recently, the study of task-switching processing has become an area of intense research that has included investigations into task switching costs, the cognitive processing involved in task switching and the neurophysiological mechanism of task switching (Kray and Feher, 2017; Kuper et al., 2017; Gaál and Czigler, 2018; Koch et al., 2018; Strobach et al., 2018; Zhao et al., 2020). In particular, studies have used transcranial direct current stimulation (tDCS) to investigate the causal relationships between activities in the dorsolateral prefrontal cortex (DLPFC) and task switching (Leite et al., 2013; Strobach and Antonenko, 2016; Strobach et al., 2016; Koch et al., 2018; Olfers and Band, 2018; Wang et al., 2020). In a previous study from our laboratory, we undertook a study in which participants were divided into three tDCS groups (left anode, right anode, and sham) to investigate anodal-tDCS (a-tDCS) induced effects on predictable and unpredictable tasks over the DLPFC (Wang et al., 2020). We showed that a-tDCS over the right DLPFC improved the switch-cost performance of unpredictable tasks (i.e., parity/magnitude tasks) and we identified a causal relationship between the right DLPFC and task switching. However, other studies have not demonstrated significant effects between right DLPFC and task switching (Strobach et al., 2018). In addition, some studies have used cross-hemispheric tDCS to show that left anode right cathode tDCS increases letter/digit naming task performance and decreases parity/vowel-consonant task performance (e.g., switch cost), and left cathode right anode tDCS has also been shown to improve accuracy in letter/digit naming tasks (Leite et al., 2013). Based on these previous studies, we hypothesize that the reasons for inconclusive results defining the relationships between right DLPFC and task switching may be due to different tasks being selected during different experiments. The exact nature of a-tDCS-induced effects over the right DLPFC in different switching tasks remains to be fully investigated. The current study aimed to analyze the roles of task characteristics under a-tDCS stimulation.

### Task-Specific Effects

We refer to the phenomenon of different tasks bringing different results as task-specific effects. In an early study on the task-specific effect of tDCS in task switching, Leite et al. (2011) tDCS (1 mA, 15 min) was applied over left DLPFC in both cognitive (i.e., color/shape switching) and motor task switching. Results have shown that a-tDCS over DLPFC increased switch-cost performance in cognitive task switching. In contrast, applying cathodal-tDCS (c-tDCS) over DLPFC significantly decreased performance on motor task switching. These data suggest the task-specific effect between cognitive and motor tasks may stem from different degrees of coactivation in motor and executive areas. In a subsequent study, the same authors stimulated DLPFC, using cross-hemispheric tDCS (1 mA, 20 min) to test effects on letter/digit naming and parity/vowel-consonant tasks (Leite et al., 2013). The results showed that left anode right cathode tDCS increased the switch-cost performance in letter/digit naming task, and the overall accuracy in parity/vowel-consonant task, but decreased the switch-cost performance in parity/vowel-consonant task. Also, left cathode right anode tDCS increased the overall accuracy in letter/digit naming tasks indicating a task-specific effect. These data suggested that different working memory loads might involve in two tasks, and in the parity/vowel-consonant task over-interpret information may occur due to more demanding situations leading to slower performance but increased accuracy. Although the left DLPFC is thought to be more associated with task switching, Wang et al. (2020) showed a specific positive role between right DLPFC and task switching. These data suggested that different cognitive control processes are involved in different switching tasks and lead to different tDCS-induced results such as irrelevant task-set inhibition, relevant task-set priming and updating (Jamadar et al., 2015). As right PFC is related to the inhibition of inappropriate responses or task-sets (Aron et al., 2004; Goel et al., 2007; Leite et al., 2013), active right DLPFC may affect parity/magnitude task performance through irrelevant task-set inhibition. Overall, this suggests that different tasks mediate the effect of tDCS. In this study, we investigated the role of the right DLPFC and a-tDCS induced effects in two different tasks, specifically a parity/magnitude task and a parity/vowel-consonant task.

### Task-Set Features

The parity/magnitude and parity/vowel-consonant tasks have been widely used to study task switching (Zhang et al., 2012; Jamadar et al., 2015; Kray and Feher, 2017; von Bastian and Druey, 2017). Based on the previous findings of Kleinsorge (Kleinsorge and Heuer, 1999; Kleinsorge, 2006), von Bastian and Druey (2017) suggested task sets have five features; judgment, dimension, mapping, response, and stimulus setting. Accordingly, we analyzed the features of these two tasks (Table 1). Task-set features involved in the parity/vowel-consonant task were greater than in the parity-magnitude task. Specifically, the parity-magnitude task mainly involved switching at the level of judgment and the parity/vowel-consonant task involved judgment and dimension levels. At the same time, stimulus sets in the parity/vowel-consonant task were the combination of eight digits and eight letters, whilst stimulus sets in the parity-magnitude task include only eight digits. This indicated that the parity/vowel-consonant task was more difficult than the parity-magnitude task. From the inhibition perspective, interference and conflict create an inhibition demand (Druey and Hübner, 2007; Jost et al., 2017; Attila et al., 2018; Ballesio et al., 2018). The parity/vowel-consonant task may also involve more inhibition in demanding situations because of interference and conflict caused by the joining of letters. A previous study showed that the right DLPFC helps in parity/magnitude tasks (Wang et al., 2020). In this study, we further explored the role of the right DLPFC in more difficult tasks (i.e., parity/vowel-consonant task). Task type (parity/magnitude vs parity/vowel-consonant) was used as a between-participant variable to determine if task type can moderate a right DLPFC a-tDCS-induced effect.

**Table 1:** Features of parity-magnitude task and parity/vowel-consonant task.

	**Parity-magnitude task**		**Parity/vowel-consonant task**	
Trial	N-1	N	N-1	N
Judgment	Parity	Magnitude	Parity	Vowel-consonant task
Dimension	Digit(value)		Digit	Letter
Mapping	Even—“F”	<5—“F”	Even—“F”	Vowel—“F”
	Odd—“J”	>5—“J”	Odd—“J”	Consonant—“J”
Response	Horizontal arrow keys		Horizontal arrow keys	
Stimulus set	1,2,3,4,6,7,8,9		Combination of digits and letters, e.g., 3A	

### Task Predictability

Task-switching sequences can be either unpredictable or predictable such as in the task-cueing paradigm. In this case, cues and stimuli appear simultaneously or in the alternating runs paradigm where tasks switch every *r* trials (Monsell et al., 2003). Recently, task predictability has attracted increasing attention (Monsell et al., 2003; Andreadis and Quinlan, 2010; Zhang et al., 2012; Schroter et al., 2015; Sabah et al., 2019; Wang et al., 2020). Zhang et al. (2012) observed task-switching performance (i.e., reaction times and accuracy) in different task predictability. The study found that performance was improved for predictable rather than for unpredictable task-switching sequences. Moreover, switch costs can be largely eliminated by increasing the preparation time in predictable task-switching sequences, however, this is not the case in unpredictable task-switching sequences. Wang et al. (2020) proposed several explanations for these performance differences. Firstly, predictable task switching includes endogenous preparation and exogenous adjustment whilst unpredictable task switching relies mainly on exogenous adjustment (Rogers and Monsell, 1995; Kiesel et al., 2010; Wang et al., 2020). Secondly, the regions of the brain involved in predictable and unpredictable tasks are different (Sohn et al., 2000; Dreher et al., 2002; Kim et al., 2011, 2015). For example, Sohn et al. (2000) found that endogenous preparation was associated with the lateral prefrontal cortex (BA46/45) and the posterior parietal cortex (BA40). The exogenous adjustment may be associated with the superior prefrontal cortex (BA8) and posterior parietal cortex (BA39/40). Also, the brain reigns involved in exogenous adjustment are different in predictable and unpredictable tasks. Previous studies have found that the role of anodal tDCS over right DLPFC is different between predictable and unpredictable tasks (Wang et al., 2020). Task predictability can be considered as an independent variable that is be expected to influence the right DLPFC a-tDCS-induced effect.

In summary, the current study aims to manipulate task predictability and task type to investigate task-switching performance during the activation of the right DLPFC and to explore a-tDCS induced and task-specific effects.

## Materials and Methods

### Study Design

The study used a 2 × 2 × 2 mixed design with task predictability (predictable, unpredictable) as a within-participant variable, tDCS group (sham, RA) and task type (parity/magnitude, parity/vowel-consonant) were between-participant variables. The dependent variables were accuracy and reaction time. Reaction times were used to calculate mixing and switch costs.

### Participants

Forty-eight undergraduate students were recruited from the Shaanxi Normal University (SNNU). The subjects had a mean age of 18.96 (*SD* = 1.458) and consisted of 41 females and 7 males. The 48 participants were randomly assigned into four groups as follows; the RA parity/magnitude task group with a mean age of 19.42 (*SD* = 1.730), the sham parity/magnitude task group with a mean age of 19.33 (*SD* = 1.577), the RA parity/vowel-consonant task group with a mean age of 18.25 (*SD* = 1.215), and the sham parity/vowel-consonant task group with a mean age of 18.83 (*SD* = 1.115). M/F ratio in every group was 2/10 except the sham parity/magnitude task group which was 1/10. All participants were right-handed, had a normal or corrected-to-normal vision and had no metallic implants or history of neurological impairment or psychiatric illness. The subjects were blinded to the purpose of this experiment and all subjects participated in the study under written informed consent.

### Stimuli and Tasks

For the predictable task, an alternating-runs paradigm required participants to shift between Tasks A and B every two trials (Kray and Feher, 2017). For the unpredictable task, a task-cueing paradigm was used that required participants to shift between Tasks A and B when a visual cue appeared (Tayeb and Lavidor, 2016). The cue was a blue background for Task A and a gray background for Task B. The task-cueing was varied in a pseudo-random way according to the previously reported technique of Wang et al. (2020). Each block had 16 switch trials and 16 repeated trials. Tasks A and B each occurred 16 times. The same stimuli did not occur continuously and there were no more than three consecutive switch trials or repeated trials.

In the parity/magnitude task, Task A required participants to indicate with a button press whether a single-presented digit was greater or smaller than 5 (by pressing “F” with the left index finger if smaller and pressing “J” with the right index finger if greater). Task B required participants to indicate with a button press whether the single-presented digit was an odd or even number (by pressing “F” if odd, and pressing “J” if even). In the parity/vowel-consonant task, Task A required participants to make the same odd/even judgment but for a stimulus that contained a number and a letter (e.g., “7K,” or “3A”). Task B required participants to judge whether the letter was a consonant or a vowel (by pressing “F” when a vowel and by pressing “J” if a consonant).

### Procedures

At the beginning of the experiment, participants were presented with two single-task blocks with 16 practice trials and 32 single-task trials on every block. The parity/magnitude task consisted of two single-task blocks including a parity and a magnitude task. The parity/vowel-consonant task consisted of two single-task blocks including a parity and a vowel-consonant task. After the tasks, the participants were administered tDCS over the right DLPFC for 20 min with a 1.5 mA intensity (see tDCS set-up details).

When the stimulation was finished and the instrument was removed, all participants completed an unpredictable and a predictable task session with one practice block and nine experimental blocks in a mixed-single sequence (i.e., mixing-task block + single-task block + ……+ single-task block + mixing-task block) (Tayeb and Lavidor, 2016). Individuals were required to reach 100% accuracy before entering the experimental blocks. The 48 participants were randomly assigned into four groups as follows; the RA parity/magnitude task group, the sham parity/magnitude task group, the RA parity/vowel-consonant task group, and the sham parity/vowel-consonant task group. In every group, half of the subjects completed the unpredictable task first and the other half-completed the predictable task first.

**Figure 1:**
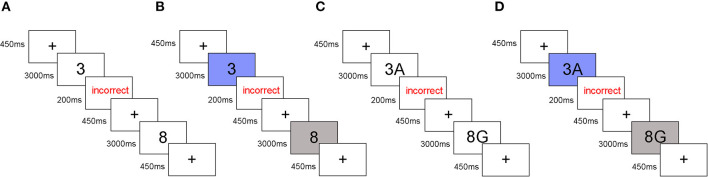
Experimental timeline of task-switching paradigm. **(A)** The predictable parity/magnitude task. **(B)** The unpredictable parity/magnitude task. **(C)** The predictable parity/vowel-consonant task. **(D)** The unpredictable parity/vowel-consonant task.

Each trial started with a 450 ms fixation point that was immediately followed by a stimulus for a maximum duration of 3,000 ms. For trials where the response was correct, the next fixation point was presented. For trials that were incorrect or had no response, feedback (the word “incorrect”) appeared for a 200 ms response followed by the next fixation point (Figure 1). The total length of the experiment was about 50–60 min including tDCS stimulation.

### tDCS Set-Up

A DC-Stimulator Plus (NeuroConn GmbH, Germany) instrument was used to deliver electrical stimulation. A pair of rubber electrodes in a 5 × 7 cm^2^ saline-soaked surface sponge was placed over the head according to the tDCS group allocation. Electrode placement was guided by the international 10–20 system (Nitsche and Paulus, 2000). For the RA tDCS group, the anode electrode was placed over F4, whilst the cathode electrode was placed on the left cheek. A 1.5 mA constant current was applied continuously for 20 min in the RA group with a linear fade in and fade out of 30 s. For the sham tDCS group, the electrodes were placed exactly as for the RA group but with an anodal pseudo-stimulation applied for 30 s over F4.

### Data Analysis

SPSS 20.0 (IBM Inc.) was used to analyze accuracy and reaction times. Before analysis of the reaction times, we excluded those with error trials, when the trial was immediately after the error trial, and the first trial of each block. Trials with reaction times exceeding 2.5 standard deviations (SD) from the mean were removed.

Several ANOVA tests were conducted on the data. We analyzed the accuracy and reaction times for the single-task blocks to confirm that the a-tDCS did not influence the performance on single trials. A 2 × 2 × 2 mixed ANOVA [single trial (pre-tDCS, post-tDCS) × tDCS group (RA, sham) × task type (parity/magnitude, parity/vowel-consonant)] was conducted.

To investigate the a-tDCS-induced effect, five separate mixed ANOVAs were conducted for the overall accuracy of mixing trials, performance (i.e., accuracy and reaction time) between switch and repeat trials, and performance between single and repeat trials. For overall accuracy of mixing trials, a 2 × 2 × 2 ANOVA [task predictability (predictable, unpredictable) × tDCS group (RA, sham) × task type (parity/magnitude, parity/vowel-consonant)] was conducted. For performance (i.e., accuracy and reaction time) between switch and repeat trials, two 2 × 2 × 2 × 2 ANOVAs [task predictability (predictable, unpredictable) × tDCS group (RA, sham) × task type (parity/magnitude, parity/vowel-consonant) × trials type (repeat, switch)] were conducted. For performance between single and repeat trials, two 2 × 2 × 2 × 2 ANOVAs [task predictability (predictable, unpredictable) × tDCS group (RA, sham) × task type (parity/magnitude, parity/vowel-consonant) × trials type (single, repeat)] were conducted.

To evaluate how activation of the right DLPFC affected the switch cost under different task types, two mixed 2 × 2 × 2 ANOVAs were conducted separately for the parity/magnitude and the parity/vowel-consonant tasks. In the parity/magnitude task, a 2 × 2 × 2 ANOVA [(task predictability (unpredictable, predictable) × tDCS group (RA, sham) × task context (parity, magnitude)] was conducted. In the parity/vowel-consonant task, a 2 × 2 × 2 ANOVA [(task predictability (unpredictable, predictable) × tDCS group (RA, sham) × task context (parity, vowel-consonant)] was conducted. To investigate the moderating effects of the task sequence, two 2 × 2 × 2 ANOVAs [(task predictability (unpredictable, predictable) × tDCS group (RA, sham) × task sequence (un-pre, pre-un)] were conducted in the parity/magnitude and the parity/vowel-consonant tasks. Please note that the data of the parity/magnitude task we used here overlaps with Wang et al. (2020).

## Results

Participants reported that they were not aware of which form of tDCS (RA or sham) they were receiving. None of the participants experienced adverse effects during or after stimulation. The descriptive statistics from the single and mixing trials are summarized in Table 2.

**Table 2:** Descriptive statistics.

**Group**	**Task type**	**Single trials**				**Mixing trials**																	
		**Pre-tDCS**		**Post-tDCS**		**Unpredictable task**									**Predictable task**								
		**ACC (%)**	**RT (ms)**	**ACC (%)**	**RT (ms)**	**ACC (%)**				**RT (ms)**					**ACC (%)**				**RT (ms)**				
						**Sin**	**Rep**	**Swi**	**Ove**	**Sin**	**Rep**	**Swi**	**MC**	**SC**	**Sin**	**Rep**	**Swi**	**Ove**	**Sin**	**Rep**	**Swi**	**MC**	**SC**
Sham	PM	95	576	96	614	96	96	94	95	602	803	1024	201	221	96	96	96	96	596	632	824	36	192
	PV	96	654	97	633	97	96	95	95	640	1038	1207	398	169	97	97	94	95	626	689	864	63	175
RA	PM	96	573	95	581	95	95	94	94	583	773	928	190	155	95	97	97	97	595	614	841	19	227
	PV	97	651	96	631	96	96	91	93	634	1015	1270	281	255	96	97	94	96	628	717	985	89	268

*Note: PM, parity/magnitude task; PV, parity/vowel-consonant task; ACC, accuracy; RT, reaction time; Sin, single; Rep, repeat; Swi, switch; Ove, overall; MC, mixing cost, referred to the differences between the single and repetition trials; SC, switch cost, referred to the differences between the switch and repetition trials*.

### Single Trials

**Figure 2:**
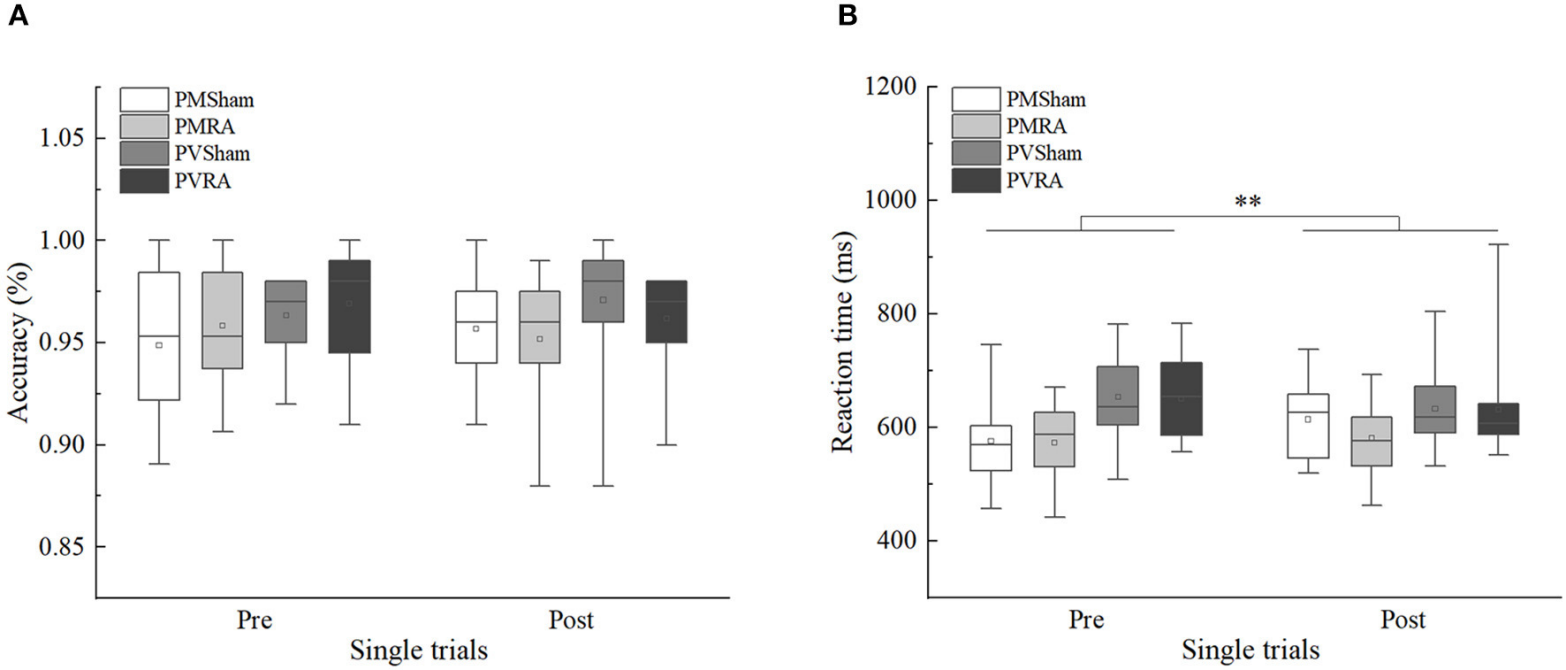
Single trials accuracy and reaction time of sham and right anode (RA) tDCS groups in pre-tDCS and post-tDCS. **(A)** Single trials accuracy. **(B)** Single trials reaction time. PMSham, single trials of parity/magnitude task in sham tDCS group; PMRA, single trials of parity/magnitude task in RA tDCS group; PVSham, single trials of parity/vowel-consonant task in sham tDCS group; PVRA, single trials of parity/vowel-consonant task in RA tDCS group. **p < 0.01.

From the mixed 2 × 2 × 2 ANOVAs (Table 3) the following results were obtained. For accuracy, no significant effects were found (*p* > 0.05). For reaction times, only the main effect of the task type was significant, *F*(1, 44) = 10.634, *p* < 0.01, η^2^*p* = 0.195, 90%CI [0.05–0.35]. Performance on the parity/vowel-consonant task (*M* = 642.42, *SE* = 11.40) was slower compared to the parity/magnitude task (*M* = 586.03, *SE* = 10.20). The main effect of the tDCS group and its related interactions did not reach significance (*p* > 0.05) (Figure 2). Moreover, we used G^*^power analysis to further define the role of the groups. For the single trial and the tDCS group, we computed the achieved power with a sample size of 48 and an effect size (f) of 0.08396038. If the probability of Type I error (α) is set as 0.05, we could reject the null hypothesis with probability [power(1-β)] as 0.989, which means that the statistical power of the sample size in the present study is reasonably high. Thus, as predicted from previous research (Tayeb and Lavidor, 2016), our findings indicate that tDCS stimulation did not generate a general tendency towards facilitation or interference.

**Table 3:** Significance statistics of single trials.

**Factors**	**Single trials ACC**		**Single trials RT**	
	***F***	***$\eta_{p}^{2}$***	***F***	***$\eta_{p}^{2}$***
ST	0.005	0.000	0.009	0.000
TG	0.002	0.000	0.341	0.008
TT	3.105	0.066	10.634**	0.195
ST × TG	2.518	0.054	0.291	0.007
ST × TT	0.005	0.000	2.550	0.055
TG × TT	0.080	0.002	0.203	0.005
ST × TG × TT	0.000	0.000	0.322	0.007

Note: ST, single trial; TG, tDCS group; TT, task type. **p < 0.01.

### Effects of tDCS on the Overall Accuracy of Mixing Trials

**Figure 3:**
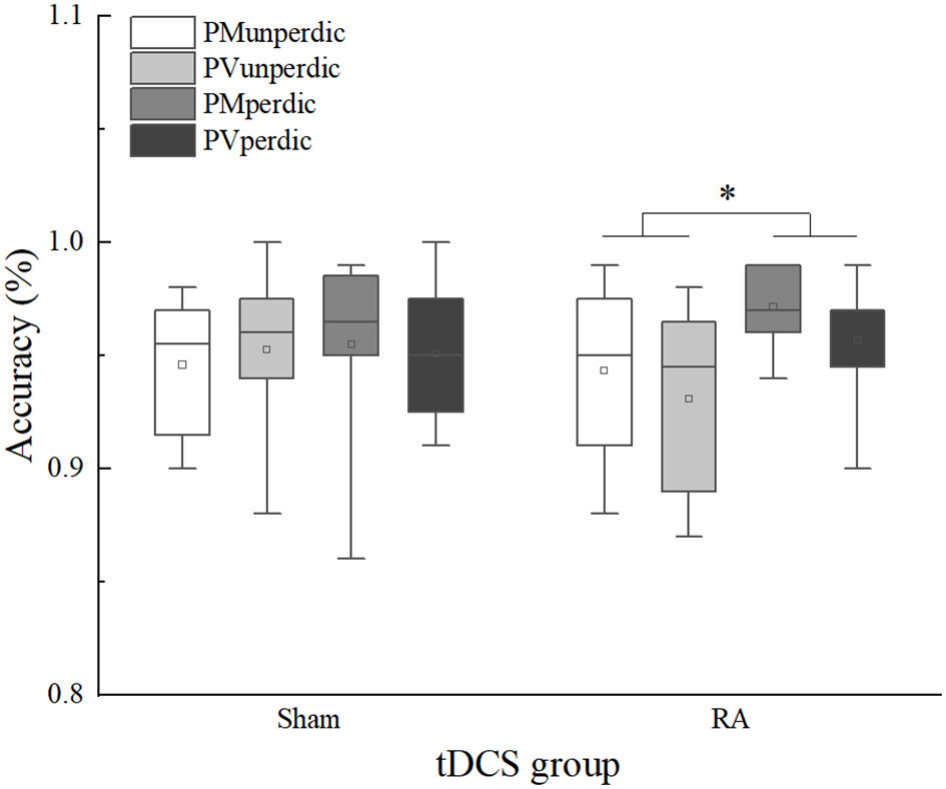
Mixing trials accuracy of sham and right anode (RA) tDCS groups in different task types and task predictability. PMunperdic, the unpredictable parity/magnitude task; PMperdic, the predictable parity/magnitude task; PVunperdic, the unpredictable parity/vowel-consonant task; PVperdic, the predictable parity/vowel-consonant task. *p < 0.05.

For the overall accuracy of the mixing trials (Table 4), the main effect of task predictability was significant, *F*(1, 44) = 8.186, *p* < 0.01, η^2^*p* = 0.157, 90%CI [0.03–0.31]. The predictable task (*M* = 0.96) was significantly more accurate than the unpredictable task (*M* = 0.94). The interaction between the tDCS group and task predictability was significant, *F*(1, 44) = 4.688, *p* < 0.05, η^2^*p* = 0.096, 90%CI [0.003–0.24]. Multiple comparisons (which were corrected using the Bonferroni method, and the threshold was set as 0.0125, which was the quotient of 0.05 divided by 4) of the interactions revealed that, in the RA group, accuracy in the predictable task (*M* = 0.96) was higher than the unpredictable task (*M* = 0.94), *F*(1, 44) = 12.633, *p* = 0.011, η^2^*p* = 0.223, 90%CI [0.06–0.38]; in the sham group, no significant difference between the predictable (*M* = 0.95) and unpredictable task (*M* = 0.95) was found, *F*(1, 44) = 0.242, *p* = 0.625, η^2^*p* = 0.005. However, no significant a-tDCS-induced effect was observed (Figure 3).

**Table 4:** Significance statistics of mixing trials.

**Factors**	**OAACC**		**SCACC**		**SCRT**		**MCACC**		**MCRT**	
	***F***	***$\eta_{p}^{2}$***	***F***	***$\eta_{p}^{2}$***	***F***	***$\eta_{p}^{2}$***	***F***	***$\eta_{p}^{2}$***	***F***	***$\eta_{p}^{2}$***
TP	8.186	0.157***	8.005	0.154**	231.59	0.084***	1.908	0.042	178.246	0.802***
TT	0.685	0.015	0.734	0.016	27.263	0.383***	1.168	0.026	20.506	0.318***
TG	0.003	0.000	0.017	0.000	0.053	0.001	0.069	0.002	0.151	0.003
TrT	–	–	36.345	0.452***	266.307	0.858***	0.224	0.005	265.767	0.858***
TP*TT	0.383	0.009	0.615	0.014	28.151	0.390***	0.049	0.001	21.751	0.331***
TP*TG	4.688	0.096*	4.106	0.085*	3.590	0.075	1.221	0.027	1.467	0.032
TP*TrT	–	–	2.187	0.047	0.895	0.020	3.508	0.074	209.418	0.826***
TT*TG	0.986	0.022	0.860	0.019	1.518	0.033	0.031	0.001	0.162	0.004
TT*TrT	–	–	7.481	0.145**	0.494	0.011	1.547	0.034	32.798	0.427***
TG*TrT	–	–	1.400	0.031	2.041	0.044	2.192	0.047	0.046	0.001
TP*TT*TG	0.149	0.003	0.271	0.006	0.014	0.000	0.003	0.000	0.170	0.004
TP*TT*TrT	–	–	1.687	0.037	0.110	0.002	0.000	0.000	19.140	0.303***
TP*TG*TrT	–	–	1.139	0.025	2.635	0.056	1.460	0.032	0.324	0.007
TT*TG*TrT	–	–	2.322	0.050	4.287	0.089*	0.014	0.000	0.188	0.004
TP*TT*TG*TrT	–	–	1.893	0.041	2.036	0.044	0.091	0.002	0.554	0.012

Note: TP, task predictability; TG, tDCS group; TT, task type; TrT, trial type; OAACC, over all accuracy of mixing trials; SCACC, accuracy between switch and repeat trials; SCRT, reaction time between switch and repeat trials; MCACC, accuracy between single and repeat trials; MCRT, reaction time between single and repeat trials. *p < 0.05; **p < 0.01; ***p < 0.001.

### Effects of tDCS on Accuracy Between Switch and Repeat Trials

**Figure 4:**
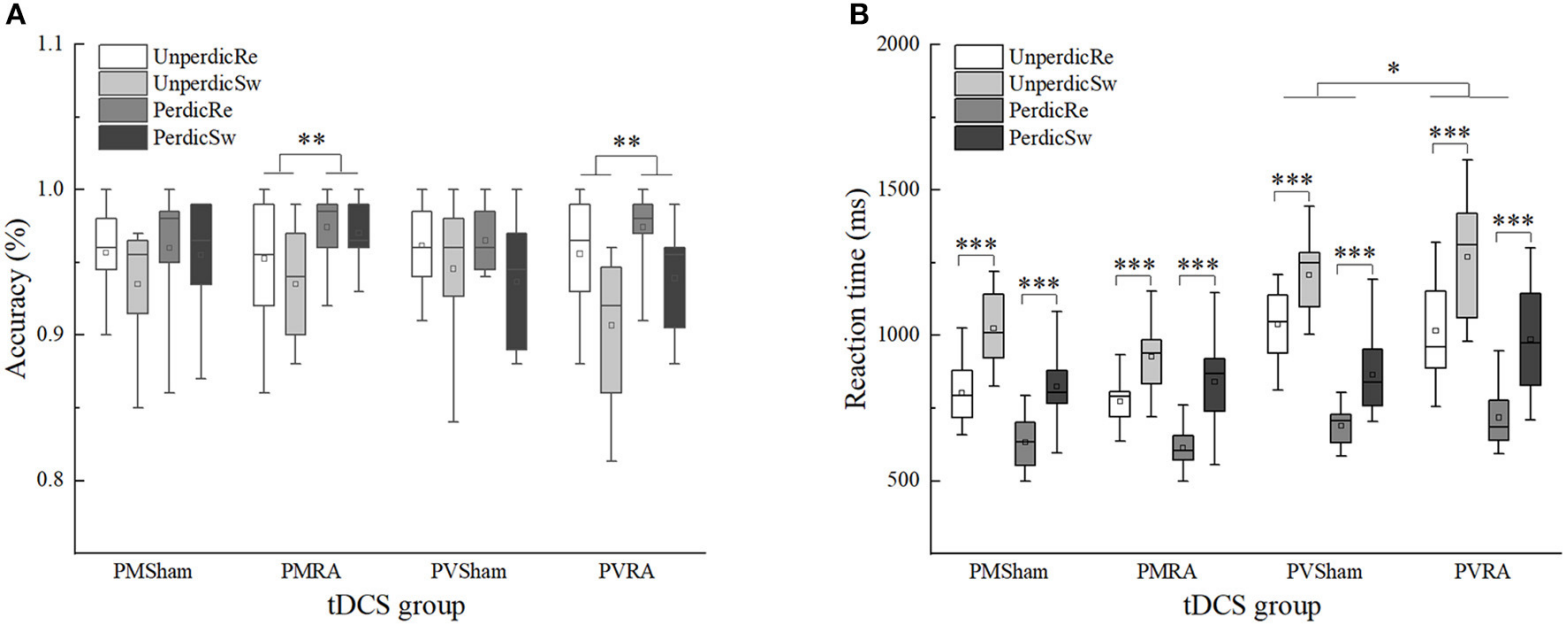
Effects of tDCS in accuracy and reaction time between switch and repeat trials in different task types and task predictability. **(A)** Effects of tDCS in accuracy between switch and repeat trials. **(B)** Effects of tDCS in reaction time between switch and repeat trials. UnperdicRe, repeat trials in unpredictable tasks; UnperdicSw, switch trials in unpredictable tasks; PerdicRe, repeat trials in predictable tasks; PerdicSw, switch trials in predictable tasks; PMSham, parity/magnitude task in sham tDCS group; PMRA, parity/magnitude task in RA tDCS group; PVSham, parity/vowel-consonant task in sham tDCS group; PVRA, parity/vowel-consonant task in RA tDCS group. *p < 0.05; **p < 0.01; ***p < 0.001.

We found a two-factor interaction between task predictability and the tDCS group, *F*(1, 44) = 4.106, *p* < 0.05, η^2^*p* = 0.085, 90%CI [0.0002–0.23]. Multiple comparisons analysis of the interactions revealed that in the RA group, the accuracy in the predictable task (*M* = 0.96) was higher than the unpredictable task (*M* = 0.94), *F*(1, 44) = 11.788, *p* = 0.001, η^2^*p* = 0.211, 90%CI [0.06–0.37]; in the sham group, no significant difference between the predictable (*M* = 0.95) and unpredictable task (*M* = 0.95) was found, *F*(1, 44) = 0.322, *p* = 0.573, η^2^*p* = 0.007. There was no significant interaction with the a-tDCS-induced effect (Table 4 and Figure 4).

### Effects of tDCS on Reaction Time Between Switch and Repeat Trials

The main effects of task predictability, trial type and task type were significant, *F*(1, 44) = 231.592, *p* < 0.001, η^2^*p* = 0.840, 90%CI [0.76–0.88]; *F*(1, 44) = 266.307, *p* < 0.001, η^2^*p* = 0.858, 90%CI [0.79–0.89]; *F*(1, 44) = 27.263, *p* < 0.001, η^2^*p* = 0.383, 90%CI [0.19–0.52]. The interaction between task predictability and task type was significant, *F*(1, 44) = 28.151, *p* < 0.001, η*2p* = 0.390, 90%CI [0.20–0.52]. The interaction between task type, tDCS group and the trials type was significant, *F*(1, 44) = 4.287, *p* < 0.05, η^2^*p* = 0.089, 90%CI [0.001–0.24]. We conducted two separate 2 × 2 ANOVAs in the different task types with the tDCS group (RA, sham) and trial type (repeated, switched). In the parity/magnitude task, only the main effect of trial type was significant, *F*(1, 22) = 156.161, *p* < 0.001, η^2^*p* = 0.877, 90%CI [0.77–0.91]. The reaction time of switch trials (*M* = 904, *SE* = 24.15) was significantly higher than repeat trials (*M* = 705, *SE* = 16.24). In the parity/vowel-consonant task, the main effect of trial type was significant, *F*(1, 22) = 118.825, *p* < 0.001, η^2^*p* = 0.844, 90%CI [0.71–0.89], and the interaction between tDCS group and trial type was significant, *F*(1, 22) = 5.021, *p* < 0.05, η^2^*p* = 0.186, 90%CI [0.01–0.39]. Multiple comparisons showed that for repeated trials, the difference between RA group (*M* = 866, *SE* = 37.50) and sham group (*M* = 864, *SE* = 22.33) was not significant, *F*(1, 22) = 0.004, *p* = 0.953, η^2^*p* = 0.000. For switched trials, the difference between the RA (*M* = 1127, *SE* = 54.81) and sham group (*M* = 1035, *SE* = 35.50) was not significant, *F*(1, 22) = 1.972, *p* = 0.174, η^2^*p* = 0.082, but with an increased reaction time (92 ms) (Table 4 and Figure 4).

### Switch Costs in the Parity/Magnitude and Parity/Vowel-Consonant Tasks

**Figure 5:**
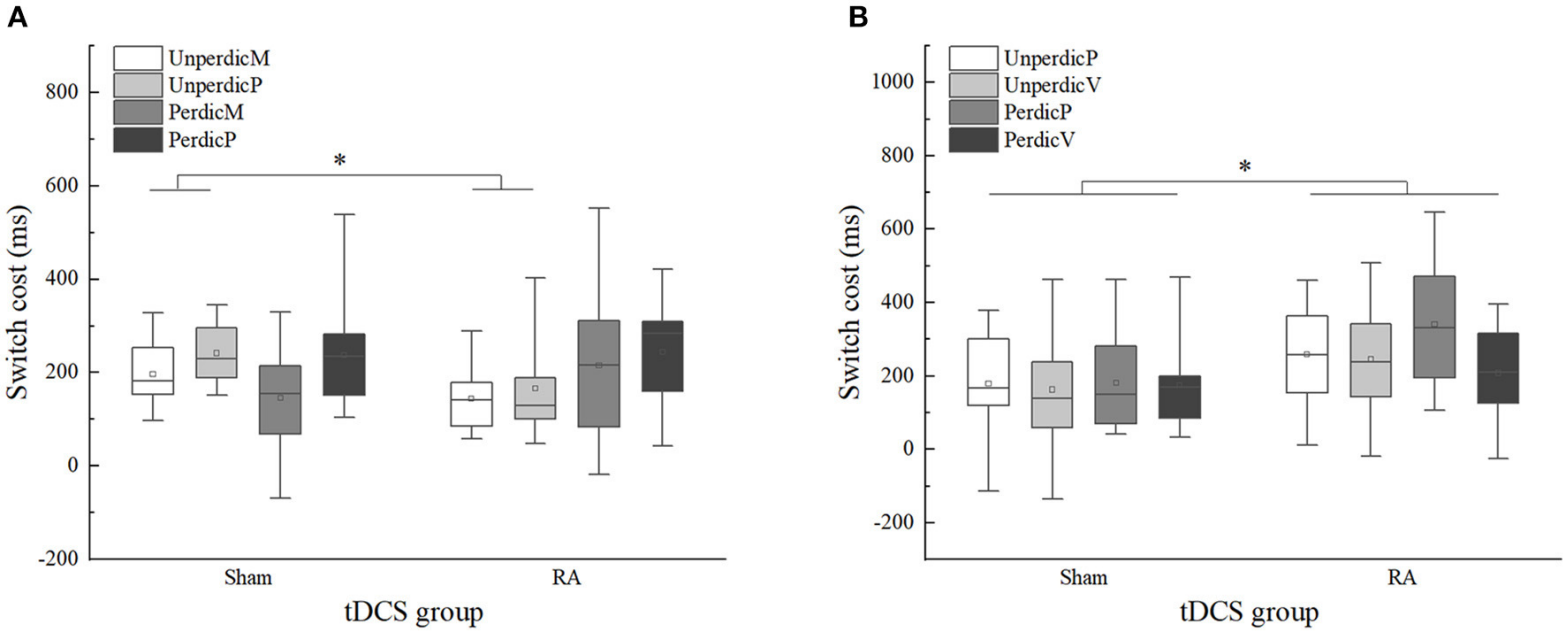
Switch cost of sham and right anode (RA) tDCS groups in the parity/magnitude and parity/vowel-consonant task in different task context. **(A)** Switch cost in the parity/magnitude task. **(B)** Switch cost in the parity/vowel-consonant task. UnperdicM, unpredictable magnitude task; UnperdicP, unpredictable parity task; UnperdicV, unpredictable vowel-consonant task; PerdicM, predictable magnitude task; PerdicP, predictable parity task; PerdicV, predictable vowel-consonant task. *p < 0.05.

To further explore how the activation of right DLPFC affected the switch-cost performance under different task types, we performed two mixed 2 × 2 × 2 ANOVA as described in the methods section (Table 5). In the parity/magnitude task, the main effect of task context was significant, *F*(*1, 22*) = 6.660, *p* < 0.05, η^2^*p* = 0.232, 90%CI [0.03–0.44]. The switching cost of the magnitude task (*M* = 175, *SE* = 19.37) was significantly lower than the parity task (*M* = 222, *SE* = 17.29). There was a significant interaction between task predictability and the tDCS group, *F*(*1, 22*) = 7.383, *p* < 0.05, η^2^*p* = 0.251, 90%CI [0.03–0.45]. Multiple comparisons showed that for the unpredictable task, the switch cost in the RA group (*M* = 155, *SE* = 22.31) was significantly lower than the sham group (*M* = 219, *SE* = 15.99), *F*(*1, 22*) =5.547, *p* = 0.028, η^2^*p* = 0.201, 90%CI [0.01–0.41]. For the predictable task, no significant difference was found between sham (*M* = 191, *SE* = 28.00) and RA group (*M* = 229, *SE* = 34.06), *F*(*1, 22*) = 0.722, *p* = 0.405, η^2^*p* = 0.032 (Figure 5). In the parity/vowel-consonant task, the main effect of the tDCS group was significant, *F*(*1, 22*) = 4.864, *p* < 0.05, η^2^*p* = 0.181, 90%CI [0.01–0.39]. The switch cost for the RA tDCS group (*M* = 263, *SE* = 29.46) was significantly higher than for the sham tDCS group (*M* = 174, *SE* = 27.90) and had no other significant effects or interactions (Figure 5).

**Table 5:** Significance statistics of switch cost in parity/magnitude and parity/vowel-consonant tasks.

**Factors**	**Task context**				**Task sequence**			
	**Parity/magnitude task**		**Parity/vowel-consonant task**		**Parity/magnitude task**		**Parity/vowel-consonant task**	
	***F***	***$\eta_{p}^{2}$***	***F***	***$\eta_{p}^{2}$***	***F***	***$\eta_{p}^{2}$***	***F***	***$\eta_{p}^{2}$***
TP	1.523	0.065	0.272	0.012	1.385	0.065	0.125	0.006
TG	0.173	0.008	4.864*	0.181	0.243	0.012	5.513	0.216*
TC	6.660*	0.232	3.059	0.122				
TS					0.273	0.013	0.317	0.016
TP × TG	7.383*	0.251	0.077	0.003	7.890	0.283*	0.013	0.001
TP × TC	0.874	0.038	1.630	0.069				
TG × TC	1.433	0.061	1.625	0.069				
TP × TS					2.813	0.123	0.035	0.002
TG × TS					0.004	0.000	3.839	0.161
TP × TG × TC	0.504	0.022	2.170	0.090				
TP × TG × TS					0.341	0.017	0.028	0.001

Note: TP, task predictability, TG, tDCS group; TC, task context; TS, task sequence. *p < 0.05.

Moreover, we analyzed the moderating effects of task sequence under different task types (Table 5). In the parity/magnitude task, we found a two-factor interaction between task predictability and the tDCS group, *F*(1, 20) = 7.890, *p* < 0.05, η^2^*p* = 0.283, 90%CI [0.04–0.49]. In the parity/vowel-consonant task, only the main effect of tDCS group was significant, *F*(1, 20) = 5.513, *p* < 0.05, η^2^*p* = 0.216, 90%CI [0.01–0.43]. These results indicated that the task sequence had no significant influence on the tDCS-induced effect.

### Effects of tDCS on Accuracy Between Single and Repeat Trials

**Figure 6:**
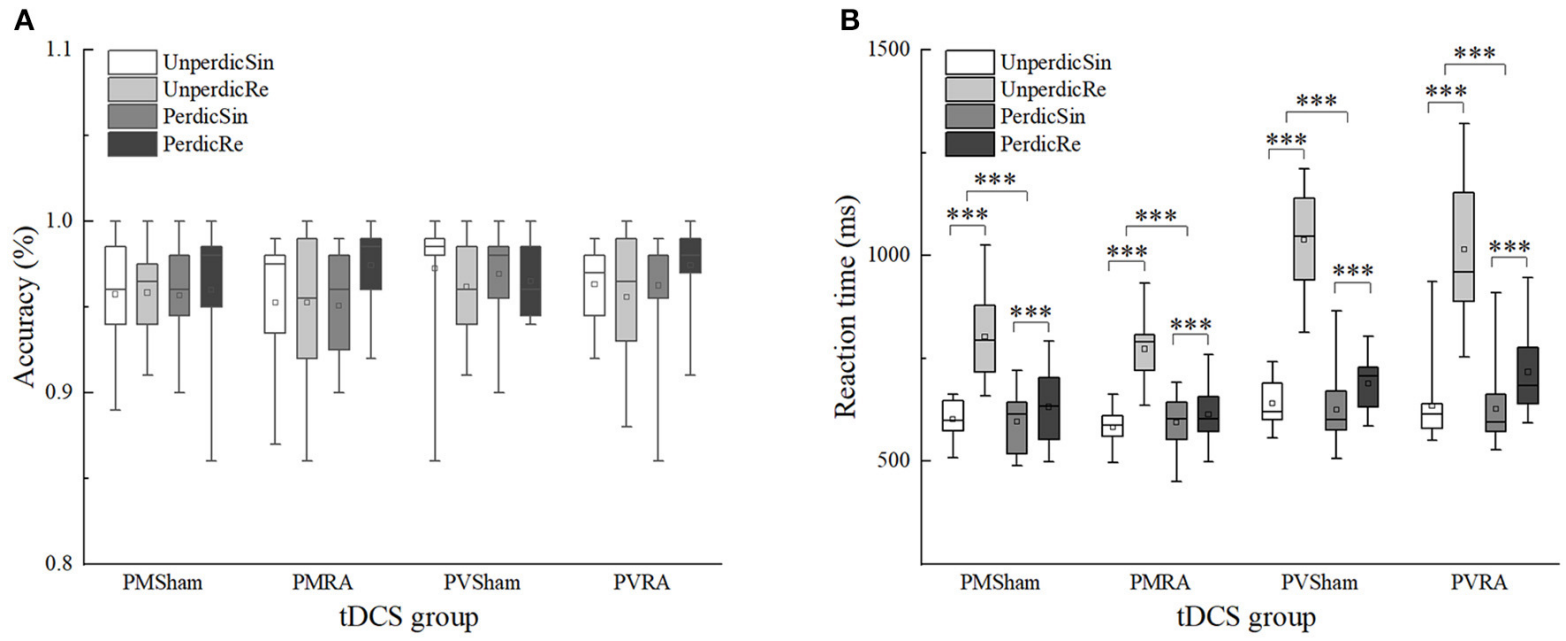
Effects of tDCS in accuracy and reaction time between single and repeat trials in different task types and task predictability. **(A)** Effects of tDCS in accuracy between switch and repeat trials. **(B)** Effects of tDCS in reaction time between switch and repeat trials. UnperdicRe, repeat trials in unpredictable tasks; UnperdicSin, single trials in unpredictable tasks; PerdicRe, repeat trials in predictable tasks; PerdicSin, single trials in predictable tasks; PMSham, parity/magnitude task in sham tDCS group; PMRA, parity/magnitude task in RA tDCS group; PVSham, parity/vowel-consonant task in sham tDCS group; PVRA, parity/vowel-consonant task in RA tDCS group. ***p < 0.001.

No significant differences in accuracy were found between single and repeat trials (*p* > 0.05) (Table 4 and Figure 6).

### Effects of tDCS on Reaction Time Between Single and Repeat Trials

The main effects of task predictability, task type and trial type were significant,

*F*(1 , 44) = 178.246, *p* < 0.001, η^2^*p* = 0.802, 90%CI [0.70–0.85]; *F*(1, 44) = 20.506, *p* < 0.001, η^2^*p* = 0.318, 90%CI [0.14–0.47]; *F*(1, 44) = 265.767, *p* < 0.001, η^2^*p* = 0.858, 90%CI [0.79–0.89]. The interaction between the task predictability and task type was significant, *F*(1, 44) = 21.751, *p* < 0.001, η^2^*p* = 0.331, 90%CI [0.15–0.48]. The interaction between task predictability and the trial type was significant, *F*(1, 44) = 209.418, *p* < 0.001, η^2^*p* = 0.826, 90%CI [0.74–0.87]. The interaction between task type and trial type was significant, *F*(1, 44) = 32.798, *p* < 0.001, η^2^*p* = 0.427, 90%CI [0.24–0.56]. The interactions between task type, task predictability and trial type were significant, *F*(1, 44) = 19.140, *p* < 0.001, η^2^*p* = 0.303, 90%CI [0.12–0.45]. However, we did not find any significant differences in the tDCS group that indicated no tDCS-induced effect on mixing cost (Table 4 and Figure 6).

## Discussion

The current study employed tDCS technology during parity/magnitude and parity/vowel-consonant tasks to investigate whether task type and task predictability would impact a-tDCS-induced effects over right DLPFC including accuracy, and the mixing and switch costs on reaction times. The primary finding was that both a-tDCS induced and task-specific effects that were observed for the switch cost but not for the mixing cost or accuracy. Specifically, in the parity/magnitude task, we demonstrated a lower switch cost for the RA group compared to the sham group for unpredictable tasks. In contrast, in the parity/vowel-consonant task, the switch cost was higher for the RA group compared to the sham group for both unpredictable and predictable tasks. These data showed an obvious opposite a-tDCS-induced effect for different types of tasks in that it was positive for the parity/magnitude tasks but negative for the parity/vowel-consonant task.

Our findings support previous studies that have aimed to change individual cognitive abilities by manipulating regions of the brain (Strobach and Antonenko, 2016; Huo et al., 2018; Nejati et al., 2018, 2020; Wang et al., 2020). Our data support the involvement of the DLPFC in task switching (Leite et al., 2011, 2013; Tayeb and Lavidor, 2016; Wang et al., 2020), specifically the right DLPFC (Vanderhasselt et al., 2006; Wang et al., 2020). Also, our results showed an opposite a-tDCS-induced effect in the different switching tasks (i.e., the parity/magnitude and the parity/vowel-consonant tasks), confirming previous research results that suggested the existence of task-specific effects, for example, the cognitive and motor tasks (Leite et al., 2011), letter/digit naming and the parity/vowel-consonant task (Leite et al., 2013).

The results from the unpredictable parity/magnitude task suggested that the anodal tDCS over right DLPFC improved task-switching performance (i.e., switch cost) in judgment levels (von Bastian and Druey, 2017). As this task only contained eight stimulus-sets, had no other switching levels and it was relatively easy to complete, the switch cost might be more due to irrelevant task-set inhibition. Except for the stimulation patterns (unilateral stimulation), the stimulation mode (offline) and in younger participants (mean age = 18.96), the right DLPFC may improve switch-cost performance by shortening the time required for the active inhibition of irrelevant information. Right DLPFC has been known to affect the inhibition ability of individuals (Aron et al., 2004). Our result suggested that the anodal tDCS over right DLPFC improved task-switching performance in judgement levels by improving the active inhibition of irrelevant information.

Our results to the predictable parity/magnitude task suggested that the anodal tDCS over right DLPFC had little impact on predictable parity/magnitude task performance (i.e., accuracy, mixing cost and switch cost). These findings confirmed a difference between the neural processes involved in performing unpredictable and predictable tasks (Monsell et al., 2003; Andreadis and Quinlan, 2010; Schroter et al., 2015; Wang et al., 2020). Unlike the unpredictable task, the predictable task allowed individuals to prepare for the next task and the longer the preparation time, the better the individual performance. From these findings, we inferred that the right DLPFC is unlikely to act on the exogenous adjustment of predictable task switching because of adequate preparation time (i.e., RSI ≥ 450 ms).

We also showed that the anodal tDCS over right DPLFC significantly decreased task-switching performance (i.e., switch cost) for the parity/vowel-consonant task in both unpredictable and predictable situations. This was not the case for the parity/magnitude task and may be due to several reasons. Firstly, because of the appearance of the letter stimulus, the parity/vowel-consonant task switching involved dimension and judgment level processes. Specifically, the stimuli in the parity/magnitude task were all digits and the participants switched between digit values (i.e., parity or magnitude). In contrast, stimuli in the parity/vowel-consonant task contained a number and a letter (e.g., “7K,” or “3A”), therefore participants switched between numbers and letters which are in two dimensions. von Bastian and Druey (2017) suggested that separate processes may be involved in the selection of judgments and stimulus dimensions. The opposite effect may reflect different processing between judgment and dimension switching which anodal tDCS over right DLPFC acted to improve judgment task-set switching and decreased stimulus dimensions switching.

Secondly, the reversal results between the parity/magnitude task and the parity/vowel-consonant task appear to be related to the phenomenon of n-2 repetition costs or backward inhibition. Considering tasks A and B, the “n-2 repeat” trial was the first trial in the task sequence, ABA. Studies have previously shown that inhibition of irrelevant tasks leads to a longer response time when the task is reactivated that is defined as the n-2 repetition costs (Koch et al., 2010; Costa and Friedrich, 2012). In the parity/vowel-consonant task, when the participants first finished the vowel-consonant task (or parity task) and switched to the parity task (or vowel-consonant task), they were required to suppress the previous task-set whilst activating and executing the current task-set. For the next switching trial, when the participants had to reactivate the vowel-consonant task (or parity task), they required longer reaction times compared to the repeat the vowel-consonant task (or parity task), or to switch to another task (e.g., magnitude). Based on our findings from the parity/magnitude task that showed anodal tDCS over the right DLPFC could improve active inhibition of irrelevant information, the results of the parity/vowel-consonant task suggested that a-tDCS over DLPFC enhanced the active inhibition of irrelevant task-set (e.g., the vowel-consonant task). However, this process unintentionally caused difficulty with reactivating the task-set leading to longer reaction times to complete the “n-2 repeat” trials. These data show that anodal tDCS over right DLPFC negatively affects n-2 repetition costs in task switching.

The n-2 repetition costs exist in the parity/vowel-consonant task and the parity/magnitude task. It is worth noting that the magnitude of the n-2 repetition cost is modulated by task difficulty (Sexton and Cooper, 2017). In a task switching paradigm, the difficulty of two tasks may vary. When the tasks are easy to finish and the task difficulty difference between them is small, the overall N-2 repetition cost (both ABA sequence and BAB sequence) is not obvious. In contrast, when the two tasks differ in difficulty, a greater N-2 repetition cost for the hard-easy-hard switches is observed compared to easy-hard-easy switches. The stronger asymmetry between tasks A and B, the more obvious the N-2 repetition costs especially in a hard-easy-hard switch manner. In the parity/magnitude task, the difficulty difference between the parity and magnitude tasks was very small and the asymmetry was not obvious. However, the difficulty difference between the parity and vowel-consonant task in the parity/ vowel-consonant task was more obvious leading to a larger asymmetry. Therefore, anodal tDCS over right DLPFC does not play a significant role in the parity/magnitude task but shows a very obvious negative effect in the parity/vowel-consonant task.

## Study Limitations

Several limitations were associated with this study. From the study design perspective, whilst the current study demonstrated a critical role for the right DLPFC in task switching, we did not investigate the underlying mechanism, especially at the dimension level of task switching. Future studies should consider combining tDCS with other technologies (e.g., ERP) to further explore which component is affected by the active right DLPFC and leads to performance changes. The influence of different a-tDCS settings on individual task performance was explored by between-subject design that may difficult to exclude the influence of individual differences on the experiment. In the future studies, within-subject design should be considered. Moreover, it is known that the wider frontoparietal network is engaged in task switching, thus, other regions of the brain should be considered to better understand the brain networks of individuals engaged in task switching such as the inferior frontal gyrus (IFG), the posterior parietal cortex (PPC), or the ventromedial prefrontal cortex (vmPFC) (Kim et al., 2011; Leite et al., 2013; Yu et al., 2015; Wang et al., 2020).

From the perspective of experimental content, as task switching involved five levels, future studies could also measure the impact of tDCS on different levels of task switching by designing more accurate tasks. However, it must be noted that the two tasks used in this study had different overall task difficulty and so it is hard to exclude the influence of this factor on the results. Further analysis of the role of task characteristics should ensure the overall difficulty of tasks are as similar as possible. Also, the parity/vowel-consonant task that we used was unlikely to be the best option for Chinese participants to study the relationship between tDCS and DLPFC in task switching of dimension levels because of the unfamiliar letters. Future studies should use a more appropriate task, for example, in the Chinese language. Finally, the participants in this study were all young college students in which the baseline level of task-switching performance is most likely higher than in other populations. This could lead to incomplete results and future studies should cover a wider range of ages to further explore the effect of tDCS on task switching performance across different age groups.

## Conclusions

From the findings presented in this study, it can be concluded that right DLPFC is involved in task switching and that active right DLPFC is most likely to influence task switching performance by changing the irrelevant task-set inhibition process. Also, right DLPFC is unlikely to act on the exogenous adjustment of predictable task switching.

## Data Availability Statement

The raw data supporting the conclusions of this article will be made available by the authors, without undue reservation.

## Ethics Statement

The studies involving human participants were reviewed and approved by ethics committee on human experimentation school of psychology, Shaanxi Normal University. The patients/participants provided their written informed consent to participate in this study.

## Author Contributions

ZW was in charge of experimental design, experimental implementation, and paper writing. RZ was responsible for experimental implementation and data analysis. XY mainly conducted overall guidance and modification of experimental design and paper. All authors contributed to the article and approved the submitted version.

## Conflict of Interest

The authors declare that the research was conducted in the absence of any commercial or financial relationships that could be construed as a potential conflict of interest.
